# Aqueous humor cytokine levels are associated with the severity of visual field defects in patients with primary open-angle glaucoma

**DOI:** 10.1186/s12886-023-02875-8

**Published:** 2023-04-05

**Authors:** Zelin Yin, Yanlin Gao, Yong Tang, Xiaofeng Tian, Yuezhong Zheng, Quanhong Han

**Affiliations:** Tianjin Eye Hospital, Tianjin Key Laboratory of Ophthalmology and Vision Science, Nankai University Eye Hospital, Clinical College of Ophthalmology, Tianjin Medical University, Gansu Road, Heping District, Tianjin, 300020 P.R. China

**Keywords:** Tumor necrosis factor-alpha, Interleukin, Transforming growth factor-beta2, Intraocular pressure, Primary open-angle glaucoma

## Abstract

**Background:**

To evaluate the aqueous humor (AH) levels of cytokines in primary open-angle glaucoma (POAG) patients and cataract patients.

**Methods:**

Thirty-eight POAG patients and 26 cataract patients were recruited. Peripheral blood (PB) was collected from each subject. The POAG group was divided into 2 subgroups according to the severity of visual field defects. The cutoff point of the mean deviation (MD) of the visual field was -12 dB. AH was obtained at the time of anterior chamber puncture during cataract or glaucoma surgery by using a 27-gauge needle attached to a microsyringe. AH and PB levels of interleukin-2 (IL-2), tumor necrosis factor-alpha (TNF-α), transforming growth factor-beta2 (TGF-β2) and IL-4 were assayed by enzyme-linked immunosorbent assay. Postoperative intraocular pressures (IOPs) of POAG patients were recorded during the follow-up period.

**Results:**

TNF-α and TGF-β2 showed significantly higher AH levels in the POAG group than in the cataract group (P < 0.001 and P = 0.001, respectively). For the POAG group, preoperative IOPs were significantly positively correlated with AH levels of TNF-α (r^2^ = 0.129, P = 0.027) and TGF-β2 (r^2^ = 0.273, P = 0.001). AH levels of TGF-β2 were significantly different among cataract patients, POAG patients with MD> -12 dB and POAG patients with MD≤ -12 dB (P = 0.001). AH levels of TNF-α were significantly positively associated with IOP reduction after trabeculectomy (P = 0.025). AH and PB levels of cytokines were not related to the long-term success of trabeculectomy.

**Conclusion:**

The levels of TNF-α and TGF-β2 showed different profiles in POAG patients and cataract patients. AH levels of TGF-β2 were correlated with the severity of glaucomatous neuropathy in POAG patients. The findings suggest possible roles for cytokines in the pathogenesis and development of POAG.

## Background

Primary open-angle glaucoma (POAG) is a progressive optic nerve degeneration characterized by continuous loss of retinal ganglion cells (RGCs) [1]. Elevated intraocular pressure (IOP) is the main risk factor for the progression of glaucoma [2]. Treatment of glaucoma focuses on IOP reduction. Medical therapy is presently the most common initial intervention to lower IOP. However, surgical management may be chosen due to inadequate IOP reduction, progression of optic nerve degeneration or visual field damage despite medical and laser treatment. Currently, trabeculectomy is a major surgical procedure to lower IOP [3].

Some cytokines have been shown in previous studies to be related to elevated IOP and glaucomatous neuropathy [4–7]. Interleukin-2 (IL-2) can increase RGC survival after optic nerve axotomy [8]. IL-4 and transforming growth factor-beta (TGF-β) inhibit nitric oxide synthesis by microglia and astrocytes in culture [9]. Furthermore, IL-4 protects RGCs from the peroxynitrite formation that results from nitric oxide synthesis by activated glial cells after central nervous system injury [9]. Tumor necrosis factor-alpha (TNF-α) is secreted in response to a variety of neuronal injuries [10, 11]. Increased expression of TNF-α and TNF-α receptor 1 indicates roles in tissue remodeling and degenerative changes in the glaucomatous optic nerve head [12]. TNF-α and its receptor-1 are upregulated in glaucomatous retinas [13]. Moreover, TNF-α-mediated cell death may lead to neurodegeneration in glaucoma [13]. These studies demonstrate that IL-2, IL-4, TGF-β2 and TNF-α levels are involved in the pathophysiological process of glaucoma and influence its initiation and development.

Excessive scarring and tissue fibrosis are major impediments to IOP reduction and the functionality of the filtering bleb after trabeculectomy [14]. Anti-TGF-β2 treatment affects surgical outcomes and suppresses conjunctival scarring [15]. TNF can stimulate the proliferation of Tenon’s capsule fibroblasts in vitro [16]. IL-4 might augment or enhance both conjunctival inflammatory and subsequent fibrotic responses in ocular cicatricial pemphigoid [17]. Moreover, serum-derived factors may change the microenvironment of the anterior chamber and the ocular cell response to cytokines in the aqueous humor. In vivo serum influx through a compromised blood-ocular barrier influences the responses of ocular cells to cytokines in the aqueous humor [18, 19]. Therefore, we simultaneously compared serum and aqueous levels of TNF-α, IL-2, TGF-β2 and IL-4 and investigated the possible effects of cytokine levels on clinical characteristics and postoperative outcomes in glaucoma patients.

## Methods

### Subjects

This study adhered to the tenets of the Declaration of Helsinki and was approved by the Ethics Committee of Tianjin Eye Hospital. Written informed consent was obtained from all subjects. All methods were carried out in accordance with relevant guidelines and regulations. The subjects were diagnosed in the Tianjin Eye Hospital, Tianjin, China. All subjects had no history of ocular surgery or ocular trauma. Patients with systemic disease affecting the levels of cytokines were excluded.

Primary open-angle glaucoma (POAG) is characterized by adult onset, IOP above 21 mmHg, glaucomatous visual field defects and optic disc damage, an open angle of normal appearance, and absence of secondary causes for glaucomatous optic disc damage [1, 3]. Topical medical therapy is presently the most common initial intervention to lower IOP, and prostaglandin analogs were selected as the initial medical therapy. If prostaglandin analogs failed to reduce IOP sufficiently, carteolol, brinzolamide, apraclonidine or acetazolamide was added to attain the desired IOP level [3]. The number of antiglaucoma medications was recorded. IOP values were ≥ 21 mmHg during follow-up before surgery. Trabeculectomy was performed when medications were insufficient to control the IOP and visual field defect.

The cataract patients had no signs of other ophthalmic diseases on complete ophthalmic examination and no family history of glaucoma. Cataract patients with high myopia were excluded. IOP values ranged from 8 mmHg to 21 mmHg before surgery.

All subjects underwent a complete ophthalmic examination, including central corneal thickness (CCT), axial length, and anterior chamber depth (ACD) measurements (Lenstar LS 900® optical biometer, Haag-Streit AG, Koeniz, Switzerland). In addition, the POAG patients underwent visual field tests using the Humphrey Field Analyzer 750i (30–2 program) (Carl Zeiss Meditec, Inc., Dublin, CA, USA). The POAG group was divided into 2 subgroups according to the severity of visual field defects. The cutoff point for the mean deviation (MD) was − 12 dB [7]. The POAG patients with an MD better than − 12 dB (MD> -12 dB) composed one group, and POAG patients with an MD worse than − 12 dB (MD≤ -12 dB) composed the other group.

## Sample collection and determination of cytokines

Three milliliters of peripheral blood (PB) were collected from each subject. Serum was then isolated and stored at − 80 °C. Aqueous humor (AH) samples were prospectively collected from 38 POAG eyes and 26 cataract eyes. Approximately 0.1 ml of AH was obtained at the time of anterior chamber puncture during cataract or glaucoma surgery; a 27-gauge needle attached to a microsyringe was used. The samples were placed in Eppendorf tubes and stored at − 80 °C until analysis.

Prior to assay, the thawed samples were centrifuged at 4 °C to remove potential debris. Levels of TNF-α, IL-2, TGF-β2 and IL-4 were determined using enzyme-linked immunosorbent assay (ELISA) according to the manufacturer’s instructions (RapidBio Lab, Calabasas, CA, USA). The suggested dilution for a normal sample is 2-fold. Each sample was measured in triplicate.

## Surgical procedure of trabeculectomy

All trabeculectomy procedures were performed by one experienced surgeon. The trabeculectomy procedure included the creation of a fornix-based conjunctival flap. After the creation of a 4 × 4 mm, half-thickness scleral flap, small pieces of surgical sponge soaked in 0.2 mg/ml mitomycin C were inserted under the conjunctival flap for 3 min. The scleral and conjunctival flaps were sutured with 10–0 nylon sutures.

## Criteria for successful/failed trabeculectomy

Preoperative and postoperative IOPs were measured using a Goldmann applanation tonometer (Carl Zeiss, Inc., Jena, Germany) on patients in the seated position. The IOP criteria for a successful surgery were defined as postoperative IOP < 21 mmHg and IOP reduction ≥ 20% without antiglaucoma medication. The IOP criteria for failed surgery were defined as IOP ≥ 21 mmHg, IOP reduction < 20%, need for anti-glaucoma medication, postoperative laser treatment or further incisional surgery for control of IOP.

### Statistical analysis

Patient gender, number of eyes (right/left), and successful surgery/failed surgery of the 2 groups were analyzed using the chi-square test or Fisher’s exact test. We used the Kolmogorov‒Smirnov test to confirm that age, preoperative IOP, number of antiglaucoma medications, axial length, ACD, CCT, MD, IOP at the final examination, IOP reduction, follow-up period, and AH and PB levels of cytokines were normally distributed. If the data were normally distributed, Student’s t test was used to compare the two groups. If the data did not follow a normal distribution, a Mann–Whitney U test was used. Correlations between IOP and levels of cytokines were analyzed using Pearson correlation analysis. AH levels of cytokines in the cataract group, MD> -12 dB, and MD≤ -12 dB POAG groups. were evaluated using one-way analysis of variance. Logistic regression analysis was performed to examine the contribution of background and AH levels of cytokines to risk factors for POAG. Linear regression analysis was performed to examine the contribution of background and AH levels of cytokines to preoperative IOP and long-term IOP reductions after trabeculectomy in the POAG group. The statistical analyses were performed with SPSS (version 19.0; SPSS Inc., Chicago, IL). P < 0.05 was considered statistically significant.

## Results

### Subject characteristics and levels of cytokines (Table 1)

**Table 1:** Characteristics of all subjects

Parameters	Cataract Group	POAG Group	P value
Number	26	38	-
Age (yrs)	58.5 ± 7.2	57.8 ± 8.0	0.324
Gender (Male/Female) *	12/14	22/16	0.447
No. eyes (right/left) *	10/16	20/18	0.314
Preoperative IOP (mmHg)	16.5 ± 2.5	31.6 ± 4.2	< 0.001
No. anti-glaucoma medication	-	3.4 ± 0.7	-
Axial length (mm)	23.1 ± 1.2	23.3 ± 1.1	0.180
Anterior chamber depth(mm)	3.0 ± 0.29	3.0 ± 0.34	0.928
Central corneal thickness (µm)	542.9 ± 30.1	551.5 ± 26.1	0.231
Mean deviation (dB)	-	-10.3 ± 5.1	-
IOP of final examination (mmHg)	-	18.5 ± 7.3	-
IOP reduction (mmHg)	-	13.0 ± 8.3	-
Relative IOP reduction (%)	-	40.3 ± 23.6	-
Follow-up period (months)	-	7.4 ± 0.9	-
Success rate of trabeculectomy (%)	-	71.10%	-
TNF-α AH (pg/ml)	1.91 ± 0.41	2.68 ± 0.93	< 0.001
TNF-α PB (pg/ml)	2.51 ± 0.54	3.76 ± 1.13	< 0.001
IL-2 AH (pg/ml)	1.30 ± 0.51	1.61 ± 0.90	0.087
IL-2 PB (pg/ml)**	1.79 ± 0.65	2.11 ± 0.97	0.279
TGF-β2 AH (pg/ml)	239.2 ± 75.6	311.3 ± 90.9	0.001
TGF-β2 PB (pg/ml)	414.6 ± 98.2	471.8 ± 103.9	0.031
IL-4 AH (pg/ml)	1.46 ± 0.74	1.78 ± 1.02	0.152
IL-4 PB (pg/ml)	1.70 ± 0.76	2.02 ± 0.96	0.138

Data are expressed as the mean ± standard deviation. *Fisher’s exact test; **Mann‒Whitney U test; other parameters were analysed using Student’s t test. Abbreviations: POAG = primary open-angle glaucoma; IOP = intraocular pressure; AH = aqueous humor; PB = peripheral blood; TNF-α = Tumor necrosis factor-alpha; IL = interleukin; TGF-β = transforming growth factor-beta

Thirty-eight POAG patients and 26 cataract patients were recruited. The patient characteristics and levels of cytokines are shown in Table 1. The mean preoperative IOP of the POAG group (31.6 ± 4.2 mmHg) was significantly higher than that of the cataract group (16.5 ± 2.5 mmHg). The mean IOP reduction was 13.0 ± 8.3 mmHg after trabeculectomy. No significant differences in age, number of eyes (right/left), gender, axial length, ACD or CCT were found between the 2 groups.

In the POAG group, TNF-α (2.68 ± 0.93 pg/ml) showed significantly higher AH levels than in the cataract group (1.91 ± 0.41 pg/ml, P < 0.001). TGF-β2 in the POAG group (311.3 ± 90.9 pg/ml) was present in AH at significantly higher levels than in the cataract group (239.2 ± 75.6 pg/ml, P = 0.001). AH levels of IL-2 and IL-4 were not significantly different between the 2 groups.

PB TNF-α levels in the POAG group (3.76 ± 1.13 pg/ml) were significantly higher than those in the cataract group (2.51 ± 0.54 pg/ml, P < 0.001), and PB TGF-β2 in the POAG group (471.8 ± 103.9 pg/ml) was significantly higher than that in the cataract group (414.6 ± 98.2 pg/ml, P = 0.031). PB levels of IL-2 and IL-4 exhibited no significant differences between the 2 groups.

## Correlation of cytokine levels between AH and PB (Fig. 1)

**Fig. 1:**
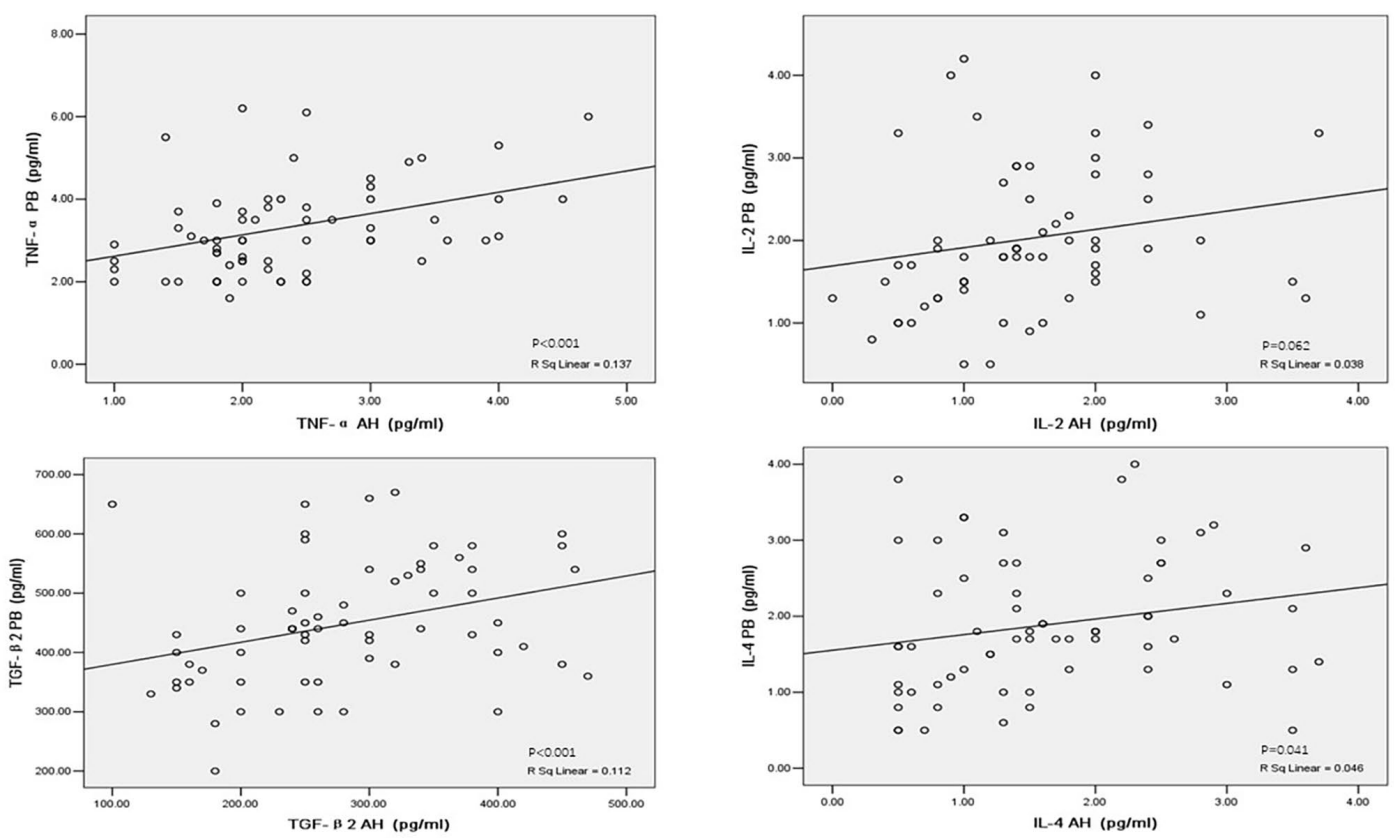
Correlation of inflammatory cytokine levels between aqueous humor and peripheral blood in all subjects (n = 64). AH levels of TNF-α, TGF-β2 and IL-4 were positively correlated with PB levels, and IL-2 showed a trend with P < 0.1. Abbreviations: TNF-α = tumor necrosis factor-alpha; IL = interleukin; TGF-β = transforming growth factor-beta; AH = aqueous humor; PB = peripheral blood

For all subjects (n = 64), AH levels of TNF-α, TGF-β2 and IL-4 correlated significantly with PB levels (2.33 ± 0.8 pg/ml vs. 3.33 ± 1.2 pg/ml, P < 0.001; 284.0 ± 95.3 pg/ml vs. 448.6 ± 106.0 pg/ml, P = 0.001; 1.69 ± 0.9 pg/ml vs. 1.90 ± 0.9 pg/ml, P = 0.041, respectively). In contrast, AH levels of IL-2 did not correlate significantly with PB levels (1.5 ± 0.7 pg/ml vs. 2.0 ± 0.8 pg/ml, P = 0.062) but showed a trend with P < 0.1.

### Logistic Regression Analysis of Subject Characteristics and AH Levels of Cytokines as a Risk for POAG (Table 2)

**Table 2:** Univariate and multivariate logistic regression analysis of background and aqueous humor levels of cytokines as a risk factor for POAG.

Factors	Univariate analysis			Multivariate analysis	
	Odds Ratio (95% CI)	P value		Odds Ratio (95% CI)	P value
Age (yrs)	0.973 (0.922–1.027)	0.320		-	-
Gender (Male/Female)	0.848 (0.361–1.993)	0.706		-	-
Axial Length (mm)	1.209 (0.829–1.764)	0.324		-	-
Anterior Chamber Depth(mm)	0.846 (0.226–3.164)	0.804		-	-
Central Corneal Thickness (µm)	1.007 (0.991–1.022)	0.391		-	-
TNF-α AH (pg/ml)	5.034 (2.176–11.646)	< 0.001		3.985 (1.465–10.844)	0.007
IL-2 AH (pg/ml)	1.637 (0.899–2.980)	0.107		0.794 (0.358–1.760)	0.570
TGF-β2 AH (pg/ml)	1.010 (1.004–1.015)	< 0.001		1.005 (0.998–1.011)	0.191
IL-4 AH (pg/ml)	1.476 (0.918–2.372)	0.108		1.013 (0.544–1.885)	0.968

Abbreviations: POAG = primary open-angle glaucoma; CI = confidence interval; AH = aqueous humor; TNF-α = tumor necrosis factor-alpha; IL = interleukin; TGF-β = transforming growth factor-beta

Univariate logistic regression analysis was performed with age, gender, axial length, ACDs, CCTs and AH levels of inflammatory cytokines. The parameters with P < 0.2 in univariate analysis were then analyzed using multivariate logistic regression analysis. Overall, the AH level of TNF-α (P = 0.007) was significantly associated with POAG (Table 2).

### Correlation between preoperative IOP and AH levels of cytokines (Fig. 2)

**Fig. 2:**
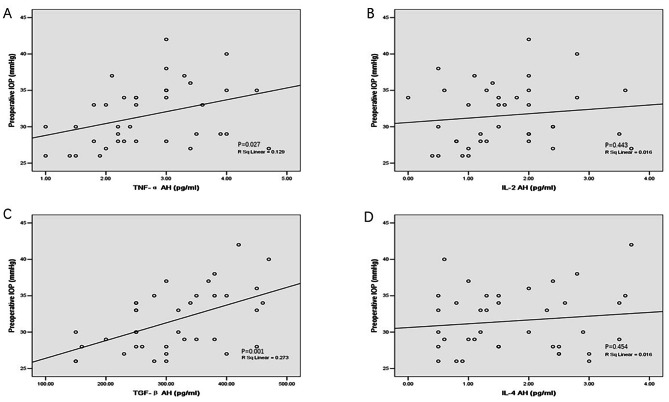
Correlation between preoperative IOP and AH levels of cytokines in the POAG group (n = 38). AH levels of TNF-α and TGF-β2 were correlated with preoperative IOP in the POAG group. Abbreviations: IOP = intraocular pressure; AH = aqueous humor; POAG = primary open-angle glaucoma; TNF-α = tumor necrosis factor-alpha; IL = interleukin; TGF-β = transforming growth factor-beta

For the POAG group, preoperative IOPs were significantly positively correlated with AH levels of TNF-α (r^2^ = 0.129, P = 0.027) and TGF-β2 (r^2^ = 0.273, P = 0.001). However, preoperative IOPs did not correlate significantly with AH levels of IL-2 and IL-4 (Fig. 2). For the cataract group, preoperative IOPs did not correlate with inflammatory cytokines (data not shown).

### Linear regression analysis of background and AH levels of cytokines as a risk factor for preoperative IOP in the POAG group

Linear regression analysis was performed with age, gender, axial length, ACD, CCT, and AH levels of inflammatory cytokines. The P value of the analysis of variance in the linear regression was more than 0.05. Therefore, background and AH levels of bioactive inflammatory cytokines did not correlate significantly with preoperative IOPs in the POAG group (data not shown).

### Correlation between long-term IOP reduction after trabeculectomy and AH levels of cytokines (Fig. 3)

**Fig. 3:**
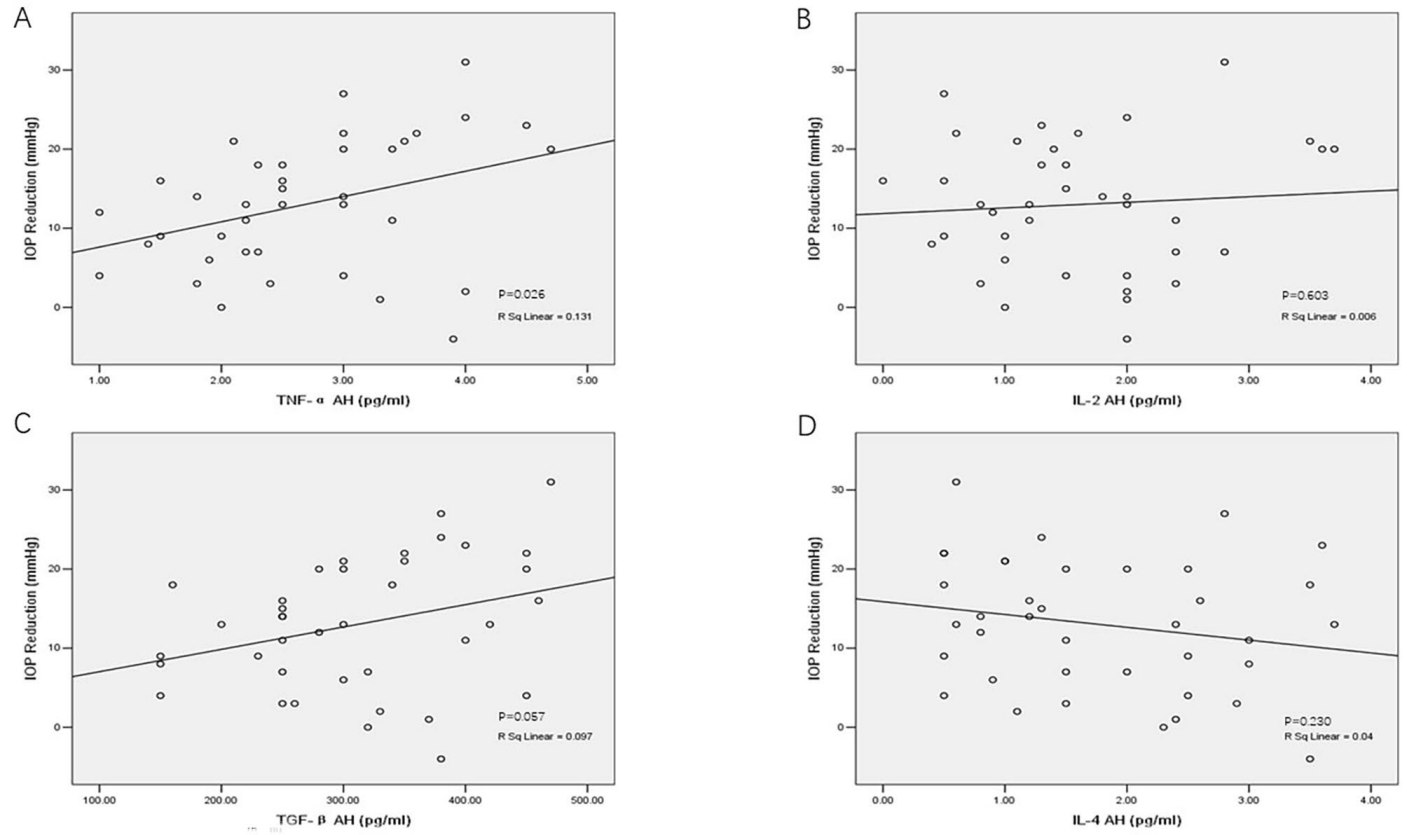
Correlation between IOP reduction and the AH levels of cytokines in the primary open-angle glaucoma group (n = 38). AH levels of TNF-α were significantly related to IOP reduction after trabeculectomy, and AH levels of TGF-β2 showed a trend with P < 0.1. Abbreviations: IOP = intraocular pressure; AH = aqueous humor; TNF-α = tumor necrosis factor-alpha; IL = interleukin; TGF-β = transforming growth factor-beta

The mean follow-up period after trabeculectomy was 7.4 ± 0.9 months in the POAG group. The mean IOP of the final examination was 18.5 ± 7.3 mmHg (Table 1). IOP at the final examination was not associated with AH levels of TNF-α, IL-2, TGF-β2 and IL-4 (P = 0.226, P = 0.937, P = 0.772 and P = 0.067, respectively). The mean long-term IOP reduction after trabeculectomy was 13.0 ± 8.3 mmHg, and the relative IOP reduction was 40.3%±23.6% (Table 1). IOP reduction after trabeculectomy significantly correlated positively with the AH level of TNF-α (r^2^ = 0.131, P = 0.026), and IOP reduction after trabeculectomy had a tendency to correlate with AH levels of TGF-β2 (r^2^ = 0.097, P = 0.057). IOP reduction after trabeculectomy did not correlate significantly with AH levels of IL-2 and IL-4 (Fig. 3).

### Linear Regression Analysis of Background and AH Levels of Cytokines as a Risk Factor for Long-term IOP Reductions after Trabeculectomy in the POAG Group (Table 3)

**Table 3:** Linear regression analysis of background and AH levels of cytokines as a risk factor for IOP reduction after trabeculectomy in the POAG group

Factors	B	95% CI for B	P value
Age (yrs)	-0.195	-0.517-0.127	0.225
Gender (Male/Female)	-2.997	-8.161-2.167	0.244
Axial length (mm)	-2.161	-4.791-0.469	0.103
Anterior chamber depth(mm)	5.434	-2.444-13.312	0.169
Central corneal thickness (µm)	-0.052	-0.157-0.054	0.325
TNF-α AH (pg/ml)	5.313	0.702-9,924	0.025
IL-2 AH (pg/ml)	-2.299	-5.640-1.042	0.170
TGF-β2 AH (pg/ml)	0.014	-0.027-0.055	0.496
IL-4 AH (pg/ml)	-2.460	-5.219-0.298	0.078

Abbreviations: AH = aqueous humor; IOP = intraocular pressure; POAG = primary open-angle glaucoma; CI = confidence interval; TNF-α = tumor necrosis factor-alpha; IL = interleukin; TGF-β = transforming growth factor-beta

Linear regression analysis was performed with age, gender, axial length, ACD, CCT and AH levels of cytokines (Table 3), and AH TNF-α levels (B = 5.313, P = 0.025) were independent risk factors for IOP reduction after trabeculectomy. Background, AH levels of IL-2, TGF-β2 and IL-4 were not significantly associated with IOP reductions after trabeculectomy.

### Correlation between AH Levels of Cytokines and Severity of Visual Field Defects in the POAG group (Table 4**and** Fig. 4)

**Table 4:** Characteristics of the POAG Group

Parameters	MD>-12 dB	MD≤ -12 dB	P value
Number	n = 27	n = 11	-
Age (yrs)	57.0 ± 8.4	57.2 ± 7.1	0.950
Gender (Male/Female) *	15/12	7/4	0.729
No. eyes (right/left) ^#^	14/13	6/5	0.880
Preoperative IOP (mmHg)	30.8 ± 4.0	33.3 ± 4.5	0.093
No. anti-glaucoma medication**	3.4 ± 0.7	3.5 ± 0.7	0.874
Axial length (mm)	23.3 ± 1.1	23.1 ± 1.1	0.480
Anterior chamber depth(mm)	2.98 ± 0.31	3.03 ± 0.40	0.557
Central corneal thickness (µm)	549.3 ± 28.5	556.6 ± 19.4	0.442
IOP of final examination (mmHg)	19.0 ± 7.1	17.5 ± 7.9	0.559
IOP reduction (mmHg)	11.8 ± 7.4	15.9 ± 10.0	0.169
Follow-up period (months)**	7.4 ± 1.0	7.4 ± 0.9	0.874
Successful surgery/Failed surgery*	19/8	8/3	1.000

Data are expressed as the mean ± standard deviation. *Fisher’s exact test; ^#^Chi-Square; **Mann‒Whitney U test; other parameters were analysed using Student’s t test. Abbreviations: POAG = primary open-angle glaucoma; MD = mean deviation; IOP = intraocular pressure

**Fig. 4:**
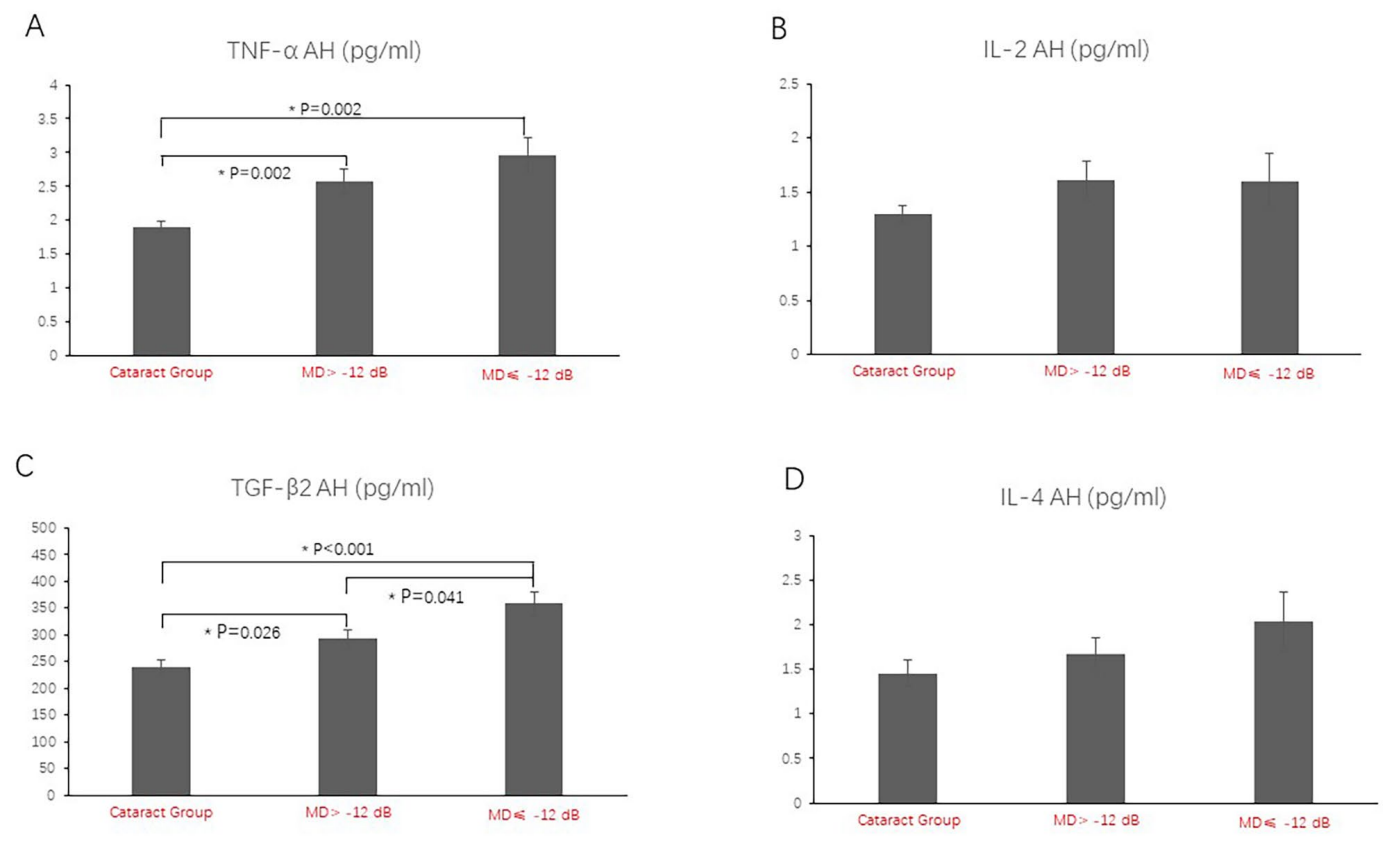
Analysis of variance of cytokines among the cataract group, MD> -12 dB, and MD≤ -12 dB POAG groups. The bar graphs show the standard error of the mean. AH levels of TNF-α and TGF-β2 were correlated with the severity of visual field defects. AH levels of IL-2 and IL-4 showed no significant difference among the groups. Abbreviations: POAG = primary open-angle glaucoma; MD = mean deviation; AH = aqueous humor; TNF-α = tumor necrosis factor-alpha; IL = interleukin; TGF-β = transforming growth factor-beta

Age, gender, number of eyes (right/left), preoperative IOP, number of antiglaucoma medications, axial length, ACD, CCT, IOP at the final examination, IOP reduction after trabeculectomy, follow-up period, and success rate (successful surgery/failed surgery) were not significantly different between the MD> -12 dB group and MD≤ -12 dB group (Table 4).

AH levels of TNF-α in the MD> -12 dB (2.57 ± 0.18 pg/ml) group and MD≤ -12 dB group (2.96 ± 0.26 pg/ml) were significantly higher than those in the cataract group (1.91 ± 0.08 pg/ml; P = 0.002, P = 0.002, respectively). AH levels of TNF-α were significantly different in the 3 groups (P < 0.001) (Fig. 4A). AH levels of TGF-β2 in the MD> -12 dB (292.22 ± 17.71 pg/ml) group and MD≤ -12 dB group (354.18 ± 21.65 pg/ml) were significantly higher than those in the cataract group (239.23 ± 14.82 pg/ml; P = 0.026, P < 0.001, respectively), and the AH level of TGF-β2 in the MD≤ -12 dB group was significantly higher than that in the MD> -12 dB group (P = 0.041). The AH levels of TGF-β2 were significantly different among the 3 groups (P = 0.001) (Fig. 4C). However, the AH levels of IL-2 and IL-4 were not significantly different among the 3 groups (Fig. 4B and D).

### Comparison of successful surgery and failed surgery in the POAG Group

Of the 38 glaucoma patients recruited, 27 underwent successful trabeculectomy, whereas failed trabeculectomy based on IOP criteria occurred in 11 cases. Age, No. eyes (right/left), preoperative IOP, No. anti-glaucoma medication, AL, ACD, CCT, MD of visual field defect, AH and PB levels of cytokines showed no significant differences between successful surgery and failed surgery (data not shown). However, female sex was a risk factor for failed trabeculectomy (P = 0.028).

## Discussion

Our study describes the AH and PB profiles of cytokines in POAG and cataract patients, and we explored possible correlations between intraocular cytokine levels and glaucomatous characteristics. In general, AH levels and PB levels of TNF-α and TGF-β2 were significantly elevated in POAG patients, and AH TNF-α and TGF-β2 levels were correlated with preoperative IOP and severity of visual field defects in the POAG group. AH levels of TNF-α were significantly related to IOP reduction after trabeculectomy, and AH levels of TGF-β2 showed a trend with P < 0.1. Nonetheless, AH levels of cytokines at the time of surgery could not predict the long-term success of trabeculectomy.

T helper 1 cells play a critical role in the regulation of cellular immunity by secreting IL-2 and TNF-α. T-helper 2 cells regulate humoral immunity by producing IL-4 and other cytokines. TGF-β can be secreted by a variety of cells to regulate cell growth and differentiation [20, 21]. AH levels of TNF-α, TGF-β2 and IL-4 were positively correlated with PB levels, and IL-2 showed a trend with P < 0.1. However, the AH levels of cytokines were lower than the PB levels in the POAG and cataract groups, which was contrary to a previous study [22]. Intraocular levels of cytokines exceeded PB levels, indicating intraocular production [22]. Therefore, further studies are needed to clarify the source of cytokines in AH and the possible mechanism leading to different levels between PB and AH.

Aqueous humor and PB levels of TNF-α were significantly higher in the POAG group. Furthermore, the AH level of TNF-α was significantly correlated with IOP reduction after trabeculectomy. However, TNF-α was not significantly different between the MD> -12 dB group and the MD≤ -12 dB group. Further studies are needed to analyze the relationship between the AH level of TNF-α and the severity of visual field defects. Ischemic or pressure-loaded glial cells can produce TNF-α, which results in oligodendrocyte death and subsequent apoptosis of RGCs [23]. Khalef et al. reported that TNF-α in AH plays a vital role in IOP elevation in patients with POAG and pseudoexfoliation glaucoma [24]. Elevated IOP plays a key role in the progression of glaucoma. PB levels of TNF-α correlate with the severity of visual defects in glaucoma patients [7]. Tong et al. reported that intraocular levels of IL-2, IL-4 and TNF-α were not different between POAG patients and senile cataract patients. There was no significant correlation between IOP and these 3 cytokines [5], which is partly consistent with our study. In a meta-analysis, open-angle glaucoma patients appeared to have higher AH levels of TNF-α than the control group [25]. Higher AH levels of TNF-α before surgery may contribute to inflammatory processes and are associated with failure of trabeculectomy at 3 months of follow-up [26]. In contrast, AH levels of TNF-α before surgery were not correlated with trabeculectomy success in our study. This discrepancy might be caused by the different races of the study population and different follow-up periods. The AH level of TNF-α was positively related to preoperative IOP, but the AH level of TNF-α was not related to the IOP at the final examination after trabeculectomy. Therefore, it seems reasonable that the AH level of TNF-α correlated with IOP reduction after trabeculectomy.

Aqueous humor or PB levels of IL-2 and IL-4 were not correlated with preoperative IOP, glaucomatous neuropathy or success of trabeculectomy in our study. Levels of IL-2 or IL-4 in glaucoma patients are controversial in previous studies. For example, Chono et al. reported that AH levels of IL-2 and IL-4 were significantly elevated in eyes with POAG [27]. The PB level of IL-2 showed no significant difference between African American healthy subjects and POAG patients [28]. PB levels of IL-2 were similar in control subjects, normal pressure glaucoma patients and POAG patients [29]. PB levels of IL-4 were significantly different in controls, patients with mild glaucomatous neuropathy and patients with severe glaucomatous neuropathy [7]. These inconsistent findings might be due to the different races or sample sizes of the study populations.

In this study, AH and PB levels of TGF-β2 were significantly correlated with preoperative IOPs and severity of visual field defects in the POAG group. Three isoforms, TGF-β1, TGF-β2 and TGF-β3, have been found in humans and other mammals [30]. TGF-β2 is the predominant subtype, although all three isoforms are expressed in ocular tissue [31, 32]. Because TGF-β2 is the main subtype in AH [33], we only measured its level in this study. One meta-analysis evaluated a total of eight studies that measured TGF-β2 levels in the AH of glaucomatous eyes. Total TGF-β2 levels were significantly elevated in open-angle glaucoma eyes, whereas both total and active TGF-β2 levels in POAG eyes were significantly higher than those in controls [4], which is consistent with our study. Release of TGF-β causes a profibrotic effect by stimulating fibroblast migration, proliferation, synthesis of collagen, and differentiation of fibroblasts into myofibroblasts [34]. Hence, a reduction in TGF-β might predict the short-term success of trabeculectomy. TGF-β2 is a key player contributing to structural changes in the extracellular matrix of the trabecular meshwork and optic nerve head, as characteristically seen in POAG [35]. Most POAG eyes with favorable bleb development show normal TGF-β2 levels, indicating a possible relationship between bleb formation and TGF-β2 levels [36]. These findings indicate that TGF-β is the main cause of excessive scarring and tissue fibrosis after trabeculectomy. In this study, AH levels of TGF-β2 were not correlated with long-term trabeculectomy success but showed a trend of correlation with IOP reduction after trabeculectomy (P = 0.057). Furthermore, AH and PB levels of cytokines were not related to the long-term success of trabeculectomy in this study. The AH level of TGF-β2 was positively correlated with preoperative IOP in the POAG group, although the AH level of TGF-β2 was not related to IOP at the final examination. According to these results, it seems reasonable that the AH level of TGF-β2 is related to IOP reduction after trabeculectomy. Because of ethical limitations, we could not test the AH level of TGF-β2 after trabeculectomy, and we thus did not analyze the relationship between the morphology of filtering blebs and AH levels of TGF-β2.

There are some limitations in this study. In the POAG group, gender was significantly different between the successful trabeculectomy group and the failed trabeculectomy group, yet female sex was not a risk factor for failed trabeculectomy in previous studies [37–40]. We should enlarge the case number in each group to reduce the impact of gender differences. AH was obtained at the time of anterior chamber puncture. We could not obtain the AH after surgery, and we could not analyze the relationship between real-time AH levels of cytokines and postoperative IOP after surgery. AH or PB levels of the cytokines are also possibly influenced by the use of anti-glaucoma medication, and our study did not analyze the relationship between the cytokines and medicine used to control the symptoms of glaucoma.

## Conclusion

TNF-α and TGF-β2 levels might be correlated with elevated preoperative IOP and IOP reductions after trabeculectomy in POAG patients and play roles in POAG pathophysiologic progression. TGF-β2 may serve as biomarkers for assessing the severity of glaucomatous neuropathy. AH levels and PB levels of cytokines at the time of surgery do not predict the outcome of trabeculectomy.

## Data Availability

All the data supporting our findings are contained within the manuscript.

## List of abbreviations

RGCs: retinal ganglion cells
IOP: intraocular pressure
IL: interleukin
TGF-β: transforming growth factor-beta
TNF-α: tumor necrosis factor-alpha
POAG: primary open-angle glaucoma
CCT: central corneal thickness
ACD: anterior chamber depth
MD: mean deviation
PB: peripheral blood
AH: aqueous humor

## References

[1] Weinreb RN, Aung T, Medeiros FA (2014). The pathophysiology and treatment of glaucoma: a review. JAMA.

[2] Konstas AG, Irkec MT, Teus MA, Cvenkel B, Astakhov YS (2009). Mean intraocular pressure and progression based on corneal thickness in patients with ocular hypertension. Eye.

[3] Gedde SJ, Lind JT, Wright MM, Chen PP, Muir KW (2021). American Academy of Ophthalmology Preferred practice pattern Glaucoma panel: primary Open-Angle Glaucoma Preferred Practice Pattern®. Ophthalmology.

[4] Agarwal P, Daher AM, Agarwal R (2015). Aqueous humor TGF-β2 levels in patients with open-angle glaucoma: a meta-analysis. Mol Vis.

[5] Tong Y, Zhou YL, Zheng Y, Biswal M, Zhao PQ, Wang ZY (2017). Analyzing cytokines as biomarkers to evaluate severity of glaucoma. Int J Ophthalmol.

[6] Guo T, Guo L, Fan Y, Fang L, Wei J (2019). Aqueous humor levels of TGFβ2 and SFRP1 in different types of glaucoma. BMC Ophthalmol.

[7] Huang P, Qi Y, Xu YS, Liu J, Liao D (2010). Serum cytokine alteration is associated with optic neuropathy in human primary open angle glaucoma. J Glaucoma.

[8] Colares TG, de Figueiredo CS, de Oliveira Jesus Souza L, Dos Santos AA, Giestal-de-Araujo E (2021). Increased retinal ganglion cell survival by exogenous IL-2 depends on IL-10, dopamine D1 receptors, and classical IL-2/IL-2R signaling pathways. Neurochem Res.

[9] Koeberle PD, Gauldie J, Ball AK (2004). Effects of adenoviral-mediated gene transfer of interleukin-10, interleukin-4, and transforming growth factor-beta on the survival of axotomized retinal ganglion cells. Neuroscience.

[10] Smith JA, Das A, Ray SK, Banik NL (2012). Role of pro-inflammatory cytokines released from microglia in neurodegenerative diseases. Brain Res Bull.

[11] Liu Y, Zhou LJ, Wang J, Li D, Ren WJ (2017). TNF-α differentially regulates synaptic plasticity in the Hippocampus and spinal cord by Microglia-Dependent Mechanisms after Peripheral nerve Injury. J Neurosci.

[12] Yan X, Tezel G, Wax MB, Edward DP (2000). Matrix metalloproteinases and tumor necrosis factor alpha in glaucomatous optic nerve head. Arch Ophthalmol.

[13] Tezel G, Li LY, Patil RV, Wax MB (2001). TNF-alpha and TNF-alpha receptor-1 in the retina of normal and glaucomatous eyes. Invest Ophthalmol Vis Sci.

[14] Igarashi N, Honjo M, Aihara M (2021). Effects of mammalian target of rapamycin inhibitors on fibrosis after trabeculectomy. Exp Eye Res.

[15] Cordeiro MF, Gay JA, Khaw PT (1999). Human anti-transforming growth factor-beta2 antibody: a new glaucoma anti-scarring agent. Invest Ophthalmol Vis Sci.

[16] Cunliffe IA, Richardson PS, Rees RC, Rennie IG (1995). Effect of TNF, IL-1, and IL-6 on the proliferation of human Tenon’s capsule fibroblasts in tissue culture. Br J Ophthalmol.

[17] Razzaque MS, Ahmed BS, Foster CS, Ahmed AR (2003). Effects of IL-4 on conjunctival fibroblasts: possible role in ocular cicatricial pemphigoid. Invest Ophthalmol Vis Sci.

[18] Chen KH, Hsu WM, Lee SM (2002). Differential effects of transforming growth factor-beta2 on corneal endothelial cell proliferation-A role of serum factors. Exp Eye Res.

[19] de Andrade FA, Fiorot SH, Benchimol EI, Provenzano J, Martins VJ, Levy RA (2016). The autoimmune diseases of the eyes. Autoimmun Rev.

[20] Akdis M, Aab A, Altunbulakli C, Azkur K, Costa RA (2016). Interleukins (from IL-1 to IL-38), interferons, transforming growth factor β, and TNF-α: receptors, functions, and roles in diseases. J Allergy Clin Immunol.

[21] Wong M, Huang P, Li W, Li Y, Zhang SS, Zhang C (2015). T-helper1/T-helper2 cytokine imbalance in the iris of patients with glaucoma. PLoS ONE.

[22] Ten Berge JC, Fazil Z, van den Born I, Wolfs RCW, Schreurs MWJ (2019). Intraocular cytokine profile and autoimmune reactions in retinitis pigmentosa, age-related macular degeneration, glaucoma and cataract. Acta Ophthalmol.

[23] Tezel G, Wax MB (2000). Increased production of tumor necrosis factor-alpha by glial cells exposed to simulated ischemia or elevated hydrostatic pressure induces apoptosis in cocultured retinal ganglion cells. J Neurosci.

[24] Khalef N, Labib H, Helmy H, El Hamid MA, Moemen L, Fahmy I (2017). Levels of cytokines in the aqueous humor of eyes with primary open angle glaucoma, pseudoexfoliation glaucoma and cataract. Electron Physician.

[25] Xin X, Gao L, Wu T, Sun F (2013). Roles of tumor necrosis factor alpha gene polymorphisms, tumor necrosis factor alpha level in aqueous humor, and the risks of open angle glaucoma: a meta-analysis. Mol Vis.

[26] Cvenkel B, Kopitar AN, Ihan A. Inflammatory molecules in aqueous humour and on ocular surface and glaucoma surgery outcome. *Mediators Inflamm* 2010, 2010:939602. 10.1155/2010/939602 PMC2864908 20467456

[27] Chono I, Miyazaki D, Miyake H, Komatsu N, Ehara F (2018). High interleukin-8 level in aqueous humor is associated with poor prognosis in eyes with open angle glaucoma and neovascular glaucoma. Sci Rep.

[28] Alapati T, Sagal KM, Gudiseva HV, Pistilli M, Pyfer M (2021). Evaluating TNF-α and Interleukin-2 (IL-2) levels in African American Primary Open-Angle Glaucoma patients. Genes (Basel).

[29] Yang J, Patil RV, Yu H, Gordon M, Wax MB (2001). T cell subsets and sIL-2R/IL-2 levels in patients with glaucoma. Am J Ophthalmol.

[30] Annes JP, Munger JS, Rifkin DB (2003). Making sense of latent TGF-beta activation. J Cell Sci.

[31] Pasquale LR, Dorman-Pease ME, Lutty GA, Quigley HA, Jampel HD (1993). Immunolocalization of TGF-beta 1, TGF-beta 2, and TGF-beta 3 in the anterior segment of the human eye. Invest Ophthalmol Vis Sci.

[32] Jobling AI, Wan R, Gentle A, Bui BV, McBrien NA (2009). Retinal and choroidal TGF-beta in the tree shrew model of myopia: isoform expression, activation and effects on function. Exp Eye Res.

[33] Jampel HD, Roche N, Stark WJ, Roberts AB (1990). Transforming growth factor-beta in human aqueous humor. Curr Eye Res.

[34] Horbelt D, Denkis A, Knaus P (2012). A portrait of transforming growth factor b superfamily signalling: background matters. Int J Biochem Cell Biol.

[35] Fuchshofer R, Tamm ER (2012). The role of TGF-β in the pathogenesis of primary open-angle glaucoma. Cell Tissue Res.

[36] Picht G, Welge-Luessen U, Grehn F, Lütjen-Drecoll E (2001). Transforming growth factor beta 2 levels in the aqueous humor in different types of glaucoma and the relation to filtering bleb development. Graefes Arch Clin Exp Ophthalmol.

[37] Romero P, Hirunpatravong P, Alizadeh R, Kim EA, Nouri-Mahdavi K (2018). Trabeculectomy with Mitomycin-C: outcomes and risk factors for failure in Primary Angle-closure Glaucoma. J Glaucoma.

[38] Fontana H, Nouri-Mahdavi K, Lumba J, Ralli M, Caprioli J (2006). Trabeculectomy with mitomycin C: outcomes and risk factors for failure in phakic open-angle glaucoma. Ophthalmology.

[39] Kanaya R, Kijima R, Shinmei Y, Shinkai A, Ohguchi T et al. Surgical Outcomes of Trabeculectomy in Uveitic Glaucoma: A Long-Term, Single-Center, Retrospective Case-Control Study. *J Ophthalmol* 2021, 2021:5550776. 10.1155/2021/5550776 PMC8163556 34094594

[40] Morita K, Gao Y, Saito Y, Higashide T, Kobayashi A (2012). In vivo confocal microscopy and ultrasound biomicroscopy study of filtering blebs after trabeculectomy: limbus-based versus fornix-based conjunctival flaps. J Glaucoma.

